# Within-Host Evolution of *Burkholderia pseudomallei* during Chronic Infection of Seven Australasian Cystic Fibrosis Patients

**DOI:** 10.1128/mBio.00356-17

**Published:** 2017-04-11

**Authors:** Linda T. Viberg, Derek S. Sarovich, Timothy J. Kidd, James B. Geake, Scott C. Bell, Bart J. Currie, Erin P. Price

**Affiliations:** a Global and Tropical Health Division, Menzies School of Health Research, Charles Darwin University, Darwin, Northern Territory, Australia; b School of Chemistry and Molecular Biosciences, The University of Queensland, Brisbane, Queensland, Australia; c Centre for Experimental Medicine, Queen’s University Belfast, Belfast, Northern Ireland; d Child Health Research Centre, The University of Queensland, Brisbane, Queensland, Australia; e Department of Respiratory Medicine, The Lyell McEwin Hospital, Elizabeth Vale, South Australia, Australia; f QIMR Berghofer Medical Research Institute, Herston, Queensland, Australia; g Department of Thoracic Medicine, The Prince Charles Hospital, Chermside, Queensland, Australia; h Department of Infectious Diseases and Northern Territory Medical Program, Royal Darwin Hospital, Darwin, Northern Territory, Australia; i Centre for Animal Health Innovation, Faculty of Science, Health, Education and Engineering, University of the Sunshine Coast, Sippy Downs, Queensland, Australia; The Sanger Institute

**Keywords:** *Burkholderia pseudomallei*, antibiotic resistance, cystic fibrosis, melioidosis, parallelism, reductive evolution, whole-genome sequencing, within-host evolution

## Abstract

Cystic fibrosis (CF) is a genetic disorder characterized by progressive lung function decline. CF patients are at an increased risk of respiratory infections, including those by the environmental bacterium *Burkholderia pseudomallei*, the causative agent of melioidosis. Here, we compared the genomes of *B. pseudomallei* isolates collected between ~4 and 55 months apart from seven chronically infected CF patients. Overall, the *B. pseudomallei* strains showed evolutionary patterns similar to those of other chronic infections, including emergence of antibiotic resistance, genome reduction, and deleterious mutations in genes involved in virulence, metabolism, environmental survival, and cell wall components. We documented the first reported *B. pseudomallei* hypermutators, which were likely caused by defective MutS. Further, our study identified both known and novel molecular mechanisms conferring resistance to three of the five clinically important antibiotics for melioidosis treatment. Our report highlights the exquisite adaptability of microorganisms to long-term persistence in their environment and the ongoing challenges of antibiotic treatment in eradicating pathogens in the CF lung. Convergent evolution with other CF pathogens hints at a degree of predictability in bacterial evolution in the CF lung and potential targeted eradication of chronic CF infections in the future.

## IMPORTANCE

*Burkholderia pseudomallei*, the causative agent of melioidosis, is an environmental opportunistic bacterium that typically infects immunocompromised people and those with certain risk factors such as cystic fibrosis (CF). Patients with CF tend to develop chronic melioidosis infections, for reasons that are not well understood. This report is the first to describe *B. pseudomallei* evolution within the CF lung during chronic infection. We show that the pathways by which *B. pseudomallei* adapts to the CF lung are similar to those seen in better-studied CF pathogens such as *Pseudomonas aeruginosa*, *Staphylococcus aureus*, and *Burkholderia cepacia* complex species. Adaptations include the accumulation of antibiotic resistance, loss of nonessential genes, metabolic alterations, and virulence factor attenuation. Known and novel mechanisms of resistance to three of the five antibiotics used in melioidosis treatment were identified. Similar pathways of evolution in CF pathogens, including *B. pseudomallei*, provide exciting avenues for more-targeted treatment of chronic, recalcitrant infections.

## INTRODUCTION

Cystic fibrosis (CF) is a genetic disorder that results in impaired mucociliary clearance of inhaled microorganisms (1). The pathophysiology of the CF lung greatly increases the risk of opportunistic bacterial infections, particularly those caused by *Pseudomonas aeruginosa*, *Haemophilus influenzae*, *Staphylococcus aureus*, and various nonfermenting Gram-negative bacteria, including members of the *Burkholderia cepacia* complex (Bcc) (2).

An underrecognized respiratory pathogen in CF is *Burkholderia pseudomallei*, a Gram-negative environmental bacterium that is endemic in most tropical regions across the globe, although the full geographic distribution remains to be elucidated (3). *B. pseudomallei* causes melioidosis, a potentially fatal disease with a wide spectrum of clinical presentations (4). Diabetes, chronic pulmonary or renal disease, hazardous alcohol use, and immunosuppression increase the risk of contracting the disease upon exposure to this bacterium (5). People with CF are considered especially at risk of melioidosis, and it is recommended that they be cautioned about traveling to locations where melioidosis is endemic and advised against undertaking exposure-prone activities (6). *B. pseudomallei* is classified as a tier 1 select agent due to its association with severe progressive disease, difficulties in accurate diagnosis, intrinsic antibiotic resistance, the lack of a vaccine, and the potential for infection through aerosolization (http://www.selectagents.gov).

All known CF patients diagnosed with melioidosis have either resided in or traveled to a known region of endemicity (7–9). Melioidosis in CF cases can present in myriad forms: acute infection with rapid deterioration, chronic infection with progressive deterioration in lung function analogous to infection with Bcc species, or subclinical infection with *B. pseudomallei* (10–12). In contrast to the pattern seen with non-CF patients, chronic *B. pseudomallei* carriage appears to be more common than acute melioidosis in those with CF (7). Only two cases of chronic *B. pseudomallei* carriage in people without CF have been previously reported: a patient with chronic lung disease who was infected for 32 months before the infection was finally eradicated (13) and another with bronchiectasis who has remained infected since first being diagnosed with melioidosis in 2000 (14). The molecular basis for chronic carriage is not yet fully known, although several factors are likely involved in bacterial persistence, including rapid genetic loss of virulence factors early in infection, antibiotic resistance, expression differences in chronically infecting strains, or an unusual or altered host immune response (14).

*P. aeruginosa* is the best-studied pathogen in CF. Once *P. aeruginosa* infection has been established in the CF lung, aggressive antibiotic treatment helps to reduce symptoms but rarely clears the infection (15). Convergent evolution of *P. aeruginosa* isolates among patients with CF has been well documented and includes the development of mucoidy, small-colony variants, multidrug resistance, hypermutators, metabolic changes, and loss of motility and secretion systems (2). Chronic *S. aureus* infections in CF also show a tendency to evolve small-colony variants, multiantibiotic resistance, and hypermutator phenotypes (16). Convergent evolution has also been noted in CF infections caused by the Bcc species *B. multivorans* and *B. dolosa* (17, 18). The most comprehensive study of Bcc evolution in the CF lung conducted to date examined a *B. dolosa* outbreak involving 14 CF cases spanning 16 years. Parallel adaptive evolution was observed in genes that affected membrane composition, decreased pathogenicity, and increased antibiotic resistance (18). Higher hypermutator prevalence has been reported in CF-associated Bcc strains, although they have not been significantly associated with antibiotic resistance, unlike hypermutators in *P. aeruginosa* (19).

In the current study, we performed whole-genome sequencing (WGS) and comparative genomic analysis of sequential *B. pseudomallei* isolates from chronically infected CF patients to document within-host evolution. *B. pseudomallei* is not part of the Bcc, and this is the first description of within-host evolution of this species in patients with CF. We hypothesized that convergent evolution of *B. pseudomallei* would occur similarly to what has been observed in other species capable of causing chronic infections, including increased antibiotic resistance and virulence attenuation. We also speculated that genome reduction would occur as the pathogen adapted to life in the CF lung.

## RESULTS AND DISCUSSION

### Phylogenomic analysis shows no specific “CF lineage.”

**FIG 1:**
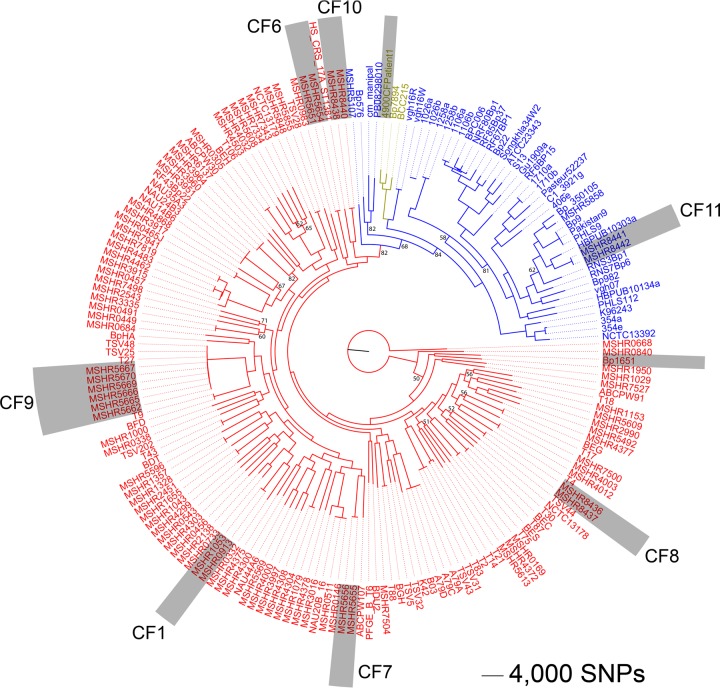
Maximum parsimony tree of 186 global *Burkholderia pseudomallei* isolates. Gray shading represents isolates from CF patients, 18 of which were sequenced in this study and 2 by others (Bp1651 [8] and 4900CFPatient1 [9]). Text color denotes geographic origin (red, Australia; blue, Asia; gold, South America). This analysis demonstrates that longitudinally collected isolates from within patients are genetically related; however, there is no distinct “CF lineage.” This analysis also confirms that CF11 was infected while traveling in Thailand; all other patients in our study acquired their infections in Australia. Branches with <85% bootstrap support are labeled. Consistency index, 0.25. Root taxon, MSHR0668.

Comparative phylogenomic analysis of the 18 CF strains against 168 publicly available *B. pseudomallei* genomes (see Table S1 and Table S2 in the supplemental material) was undertaken to assess the relatedness of the 186 strains. Phylogenetic reconstruction using 178,086 core genome biallelic single-nucleotide polymorphisms (SNPs) showed that, although *B. pseudomallei* isolates from individual patients grouped together at the extreme nodal points of the phylogeny, there was no specific “CF lineage” (Fig. 1; see also Fig. S1 in the supplemental material).

10.1128/mBio.00356-17.5 TABLE S1  List of the 186 *Burkholderia pseudomallei* genomes used for comparative phylogenomic analysis of the seven CF strain pairs. Download TABLE S1, XLSX file, 0.02 MB. Copyright © 2017 Viberg et al. 2017 Viberg et al. This content is distributed under the terms of the Creative Commons Attribution 4.0 International license.

10.1128/mBio.00356-17.6 TABLE S2  Summary of *Burkholderia pseudomallei* isolate metadata and mutational events observed in sequential isolates from CF patients. Abbreviations: SRA, sequence read archive; SNP, single-nucleotide polymorphism; indel, insertion-deletion; NS, nonsynonymous; S, synonymous. ^a^, numbers in parentheses correspond with the patient identifier (ID) as presented reference 7. ^b^, data were determined on the basis of the *B. pseudomallei* multilocus sequence typing scheme (http://pubmlst.org/bpseudomallei). ^c^, data include all SNPs, indels, and larger deletions identified by SPANDx (79) using default settings. ^d^, data were determined on the basis of patient epidemiological data and comparative phylogenomic analysis (Fig. 1). ^e^, data refer to patients suspected to have been infected ~3 years prior to the first *B. pseudomallei* isolate being obtained/available for this study. ^f^, data correspond to samples collected 2 days post-bilateral lung transplant; ^g^, data are from reference 76. Download TABLE S2, XLSX file, 0.01 MB. Copyright © 2017 Viberg et al. 2017 Viberg et al. This content is distributed under the terms of the Creative Commons Attribution 4.0 International license.

10.1128/mBio.00356-17.2 FIG S1  Maximum likelihood phylogeny of 186 global *Burkholderia pseudomallei* isolates. Root taxon, MSHR0668. Download FIG S1, JPG file, 3.3 MB. Copyright © 2017 Viberg et al. 2017 Viberg et al. This content is distributed under the terms of the Creative Commons Attribution 4.0 International license.

Unlike other CF pathogens, most of which are capable of patient-to-patient transmission, *B. pseudomallei* is almost always environmentally acquired and is rarely transmitted between humans (20) and as such demonstrates a very strong phylogeographic signal that allows accurate identification of strain origin on a continental level (21). Using this information, our analysis revealed that patient CF11 was infected while traveling in Southeast Asia, consistent with the reported travel history for this patient, whereas all other patients in our study acquired their *B. pseudomallei* infections in Australia (Fig. 1). Two *B. pseudomallei* CF isolates from other studies, Bp1651 (8) and 4900CFPatient1 (9), grouped according to their reported patient travel history of Australia and South America, respectively.

### *B. pseudomallei* parallel adaptation toward life in the CF airway.

In comparison to Bcc species, where transmission of a previously adapted strain from one individual to another is relatively common, adaptation of *B. pseudomallei* in the CF lung almost always represents independent evolution of a fully virulent wild-type (WT) strain. Mutational convergence across CF infections in our study would therefore strongly point to a degree of predictability in pathogen behavior as the population adapts to the CF lung niche, providing an unbiased approach to assess this parallel evolutionary signal.

To quantify evidence of selection and parallel evolution in our data set, we first investigated the role of intragenic bias in the paired isolates from each CF case; midpoint (i.e., intermediate) isolates collected from patient CF9 were not examined. A previous study of 112 *B. dolosa* isolates retrieved from 14 CF cases showed no bias (18). In contrast, we saw a significant (*P* < 0.01) bias against intragenic mutations in our data set, with only 110 of 170 (65%) of the observed SNP and indel mutations occurring in coding regions (expected, 143 [84%]; 99% confidence interval, 76% to 91%). This finding indicates that the *B. pseudomallei* isolates are under strong purifying selection. Next, we screened for overrepresentation of mutational events in single genes to compare the degree of parallelism to the frequency of randomly distributed events. Analyzing just SNP and indel variants, 97 genes possessed a single mutation, 5 genes (*fabG*, *gyrA*, *ptr1*, *bpeT*, and *BPSS2021*) possessed two mutations, and 1 gene (*vgrG*) possessed three mutations versus the expected rates of 136.7, 2.8, and 0.1, respectively. Inclusion of deleted and duplicated loci increased the number of genes with two mutations to 13 (including *fabF*, *BPSS0945*, *BPSS0948*, *BPSS0949*, *BPSS0950*, *BPSS0951*, *BPSS0952*, and *BPSS1632*) and the number of genes with three mutations to 2 (including *penA*). Four of these genes (*gyrA*, *ptr1*, *bpeT*, and *penA*) have been linked to antibiotic resistance, and *fabF* and *fabG* are associated with fatty acid biosynthesis. Taken together, these results reveal evidence of *B. pseudomallei* parallel evolution in the CF lung toward mutations that have a selective advantage in this environment. The role of these mutations in the CF cases is discussed in more detail in the relevant subsections below.

### Mutations in *mutS* lead to the development of hypermutator strains.

**FIG 2:**
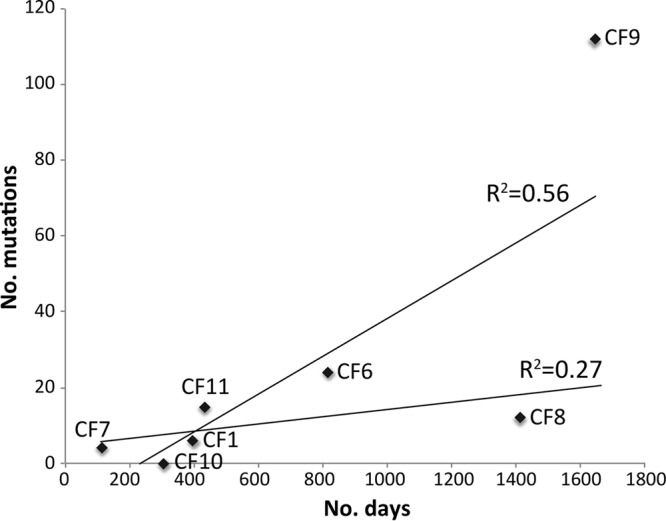
Mutations accrued in paired *Burkholderia pseudomallei* isolates over time in seven CF patients. The latter isolate from patient CF9 had a much greater number of mutations than expected in comparison to other CF isolate pairs due to defects in the DNA mismatch repair system, driving a hypermutator phenotype. The line of best fit is dramatically different when CF9 is included.

We observed that, in general, *B. pseudomallei* strains from the CF airway accumulated mutations over time at similar rates. Six (CF1, CF6, CF7, CF8, CF10, and CF11) of the seven pairs accrued mutations at a mean rate of 6.4 mutations/year (Fig. 2; see also Table S2 in the supplemental material). Examining only SNPs, this rate was 3.6 SNPs/year (4.9 × 10^−7^ substitutions/site/year), similar to those determined previously for *B. dolosa* at 2.1 SNPs/year (3.3 × 10^−7^ substitutions/site/year [18]) and for *B. multivorans* at 2.4 SNPs/year (3.6 × 10^−7^ substitutions/site/year [17]). In contrast, the CF9 pair was a clear outlier, with 24.9 mutations/year and 12.9 SNPs/year (1.8 × 10^−6^ substitutions/site/year; Fig. 2). The most recent isolate from CF9 had the highest number of mutations of all paired isolates, with 112 mutational events accrued over 55 months. As a comparison, the paired isolates from CF8, which were obtained 46 months apart, had only 12 mutational events (Table S3).

10.1128/mBio.00356-17.7 TABLE S3  Complete list of SNPs, indels, large deletions, and duplicated regions in the paired CF isolates. Download TABLE S3, XLSX file, 0.04 MB. Copyright © 2017 Viberg et al. 2017 Viberg et al. This content is distributed under the terms of the Creative Commons Attribution 4.0 International license.

Patient CF9 isolates collected at the midpoint also had an elevated number of mutations compared with the initial strain from this patient (Table S3). Although not previously documented in *B. pseudomallei*, the high mutation rate observed in CF9 isolates pointed toward the evolution of hypermutation. We identified four mutations affecting genes within the DNA mismatch repair (MMR) system, which can induce hypermutator phenotypes when rendered nonfunctional (22). Notably, *mutS* (*BPSL2252*) was mutated in all sequential isolates from CF9. The first mutation, a 1-bp insertion in *mutS*, was observed in all sequential isolates except the 33-month isolate. This insertion resulted in a frameshift at residue 354 (N354fs) of MutS, leading to truncation from 894 to 642 amino acids. The 33-month isolate instead possessed a 45.5-kb deletion that encompassed the first 158 bp of *mutS*. Both mutations were predicted to abolish MutS function *in silico* (Table S3; Table S4), suggesting strong selection toward MutS inactivation in this patient. Two other MMR system loci were also mutated in the final isolate from CF9, with a V45A substitution in the DNA polymerase/helicase locus *BPSL2556* and a 1-bp insertion 41 bp upstream of the *mutD* (*BPSL1341*) coding sequence. Due to the lack of these mutations in midpoint CF9 isolates, the contribution, if any, of these additional MMR system mutations to hypermutability in *B. pseudomallei* is unclear.

10.1128/mBio.00356-17.8 TABLE S4  Color block of SNP and indel mutations across all CF9 isolates. Download TABLE S4, XLSX file, 0.1 MB. Copyright © 2017 Viberg et al. 2017 Viberg et al. This content is distributed under the terms of the Creative Commons Attribution 4.0 International license.

Other CF pathogens such as *P. aeruginosa* can evolve distinct subpopulations that occupy different niches within the CF airway (23). This phenomenon may explain the distinct and yet convergent mutations in MMR genes that have led to the hypermutator phenotype in the CF9 isolates. To examine the issue further, phylogenies were reconstructed for all CF9 isolates using SNPs alone (see Fig. S2A and C for maximum parsimony and maximum likelihood trees, respectively) and combined SNP-indel data (Fig. S2B), as we previously demonstrated that inclusion of indels in the phylogenetic analysis of closely related *B. pseudomallei* strains provides greater phylogenetic resolution and better correlation with strain metadata than the use of SNPs alone (24, 25). This analysis identified multiple subpopulations that did not completely correspond with passage of time. For example, the 55-month isolate did not harbor the greatest number of mutations, with the 49-month isolate and the 33-month isolate having more mutations (Table S3). Paradoxically, the 33-month isolate also clustered within the *mutS* N354fs clade, despite not having this mutation (Fig. S2). It is possible that this strain either reverted to the wild-type genotype or recombined with an isolate with the wild-type genotype before accruing the deletion. Another possible scenario is that the frameshift mutation did not become fixed in the population and is therefore not seen in the 33-month isolate. Collection and WGS of multiple colonies from each clinical specimen, as described in other studies (18, 26, 27), would be needed to establish more precisely the order and prevalence of these mutational events, with our results demonstrating that the *B. pseudomallei* population in the CF9 lung is much more complex than was appreciated at the time of sampling and isolate storage.

10.1128/mBio.00356-17.3 FIG S2  Phylogenetic and root-to-tip regression analyses of all CF9 isolates. (A) Maximum parsimony using SNPs only (CI = 1.0). (B) Maximum parsimony using a combination of SNPs and indels (CI = 0.98). (C) maximum likelihood. *, a *mutS* indel that causes an N354fs mutation is encoded; #, a 45.5-kb deletion that includes the first 158 bp of *mutS* is encoded. Root taxon, MSHR5662. Download FIG S2, PDF file, 0.9 MB. Copyright © 2017 Viberg et al. 2017 Viberg et al. This content is distributed under the terms of the Creative Commons Attribution 4.0 International license.

For patients CF1, CF9, and CF11, the first isolate was collected approximately 3 years (~1,000 days) post-melioidosis diagnosis (Table S2). Using the temporal data set available for CF9, we performed a root-to-tip regression analysis to determine whether the predicted infection date in this patient reflected the probable infection date. On the basis of the SNP phylogenies, the dates were estimated at ~600 days (Fig. S2A and C) and at ~400 days for the SNP-indel phylogeny (Fig. S2B). Therefore, these calculated infection dates do not provide a reliable indication of time since infection, probably due to the emergence of hypermutator lineages in CF9 over time and the different rates of evolution among the members of the CF9 *B. pseudomallei* population. Our results demonstrate that caution should be taken in interpreting results of ancestor dating methods where hypermutation has occurred during the infection, with underestimation of the predicted infection date to be expected in such cases, particularly if different mutation rates are not taken into account.

### Evolution of multidrug-resistant infections in three CF patients.

Cases of multidrug-resistant *B. pseudomallei* (i.e., strains resistant to ≥2 clinically relevant antibiotics) are uncommon. One example is CF isolate Bp1651, which is resistant to amoxicillin-clavulanate (AMC), ceftazidime (CAZ), doxycycline, imipenem, and trimethoprim-sulfamethoxazole (TMP/SMX) (8). Due to the lengthy antibiotic treatment regimens administered to many CF patients, this cohort is at an elevated risk of developing multidrug-resistant infections. Therefore, we tested all *B. pseudomallei* strains collected for this study for resistance to the clinically relevant antibiotics CAZ, meropenem, TMP/SMX, doxycycline, and AMC (Table S5). Sensitivity to these antibiotics was noted in all isolates from patients CF1, CF7, CF8, and CF10. In patient CF7, isolates were obtained prior to treatment, and in patient CF8, no treatment was administered; thus, the development of antibiotic resistance was not expected in these patients.

10.1128/mBio.00356-17.9 TABLE S5  MICs of longitudinally collected *Burkholderia pseudomallei* isolates from chronically infected CF patients. Abbreviations: CAZ, ceftazidime; MP, meropenem; TMP/SMX, trimethoprim-sulfamethoxazole; DC, doxycycline; AMC, amoxicillin-clavulanate; CIP, ciprofloxacin; ND, not determined. Bold highlighting indicates a MIC above the CLSI cutoff values for resistance. Download TABLE S5, XLSX file, 0.01 MB. Copyright © 2017 Viberg et al. 2017 Viberg et al. This content is distributed under the terms of the Creative Commons Attribution 4.0 International license.

In comparison, certain isolates from patients CF6, CF9, and CF11 showed multidrug-resistant profiles (Table S5), consistent with these patients being treated with several rounds of antibiotics without clearing *B. pseudomallei*. For CF9, multidrug resistance to TMP/SMX and doxycycline was observed in an isolate obtained just 2 days post-bilateral lung transplant, pointing to the persistence of a multidrug-resistant population that infected the newly transplanted lungs, presumably from the upper airway. Curiously, other isolates from CF9 did not exhibit multidrug resistance despite this patient being maintained on doxycycline for *B. pseudomallei* suppression posttransplantation (7). For CF6, a multidrug-resistant isolate was obtained that was resistant to both CAZ and TMP/SMX, and for CF11, both isolates developed resistance to TMP/SMX, with resistance also to either CAZ or doxycycline. These results highlight the heightened risk of emergence of multidrug resistance in *B. pseudomallei* following administration of aggressive and prolonged antibiotic treatment regimens.

### CAZ resistance in patients CF6 and CF11 is conferred by the β-lactamase PenA.

The development of CAZ resistance in patients CF6 and CF11 was consistent with their treatment, with both patients administered multiple courses of this antibiotic to treat *P. aeruginosa* and *B. pseudomallei*. The initial isolate from patient CF11 and the latter isolate from patient CF6 demonstrated intermediate and high-level resistance to CAZ, with MIC values of 12 and ≥256 μg/ml, respectively (Table S5). CAZ resistance in patient CF6 occurred through a C69Y mutation in the β-lactamase PenA, a known mutation for high-level CAZ resistance in *B. pseudomallei* (28). In contrast, the isolate from CF11 encoded no previously known mechanisms for CAZ resistance (28–30). Closer examination of sequencing coverage depth across the paired isolates revealed that the initial CF11 strain had a 36.7-kb duplication encompassing *penA* that represented approximately 10× deeper coverage than average, with the same region in the latter strain collected from patient CF11 having twice the average depth of coverage (Fig. S3G). These duplications were confirmed by quantitative PCR (qPCR) analysis of *penA*. Only the 10× duplication produced clinically relevant resistance to CAZ; the 2× increase did not detectably increase the MIC (Table S5). Patient CF11 underwent two eradication attempts, including 84 days of CAZ treatment. It is possible that the initial CF11 strain underwent a duplication of *penA* in response to contemporaneous selection pressure, with remnants of this gene duplication still present when the latter isolate was retrieved.

10.1128/mBio.00356-17.4 FIG S3  Coverage plots of sequencing read depth across the seven paired *Burkholderia pseudomallei* isolates relative to *B. pseudomallei* K96243. Black arrows in the CF6 and CF11 pairs indicate convergent duplications that encompass the β-lactamase PenA. These duplications lead to increased resistance to the β-lactam antibiotic ceftazidime. Download FIG S3, TIF file, 5.2 MB. Copyright © 2017 Viberg et al. 2017 Viberg et al. This content is distributed under the terms of the Creative Commons Attribution 4.0 International license.

Intriguingly, in addition to the C69Y mutation, the CF6 strain had an ~30× duplication of an ~7.5-kb region that encompassed the *penA* gene (Fig. S3B). The C69Y mutation was present in all 30 copies of this locus, demonstrating that the duplication event occurred following mutation. As C69Y itself confers high-level resistance to CAZ (28), it remains unknown whether the 30× duplication event confers an additional selective advantage in this isolate. The possibility that the genetic location of the tandemly repeated loci in the CF6 and CF11 isolates was in a plasmid was ruled out based on visualization of assembly graphs using Bandage (31), which showed that these loci were chromosomally integrated and tandemly repeated, with no evidence of the presence of essential plasmid replication or maintenance machinery in those regions (data not shown). To our knowledge, gene duplication has not previously been reported as a mechanism for clinically significant antibiotic resistance in *B. pseudomallei*, although it has been observed *in vitro* in *B. thailandensis* mutants, where tandem repeat regions within the omega loop of *penA* modestly increased CAZ MICs, and the presence of full-length WT *penA* on a multicopy plasmid greatly increased the CAZ MIC in this species (32). Given that patients CF6 and CF11 ultimately died, this molecular mechanism should be considered in all cases of chronic *B. pseudomallei* infections, particularly in those patients who demonstrate no improvement in symptoms following lengthy or multiple courses of CAZ.

### Putative mechanisms conferring TMP/SMX resistance.

Isolates from patients CF6, CF9, and CF11 showed high-level (≥32 µg/ml) resistance to TMP/SMX (Table S5). Although the precise molecular mechanisms conferring TMP/SMX resistance in *B. pseudomallei* have not yet been conclusively identified, an increase in the TMP/SMX MIC has previously been associated with upregulation of the resistance-nodulation-cell division (RND) BpeEF-OprC multidrug efflux pump (33). These increases were, however, generally small, and their clinical relevance is not known (33, 34). BpeEF-OprC is under regulatory control by the LysR-type regulators BpeT (encoded by *BPSS0290*) (35) and BpeS (encoded by *BPSL2111*) (33). None of the TMP/SMX-resistant isolates in this study had mutations in *bpeS*, and only the latter patient CF6 isolate had a mutation in *bpeT*, which resulted in a frameshift at T314 and truncation of BpeT from 334 to 330 residues (Table S3). Therefore, it is unclear what role, if any, BpeT plays in TMP/SMX resistance in our CF isolates.

Previous work has shown that mutations affecting pteridine reductase 1 (Ptr1; encoded by *BPSS0039*) can increase TMP/SMX MICs in *B. pseudomallei* (33). Both TMP/SMX-resistant (MIC of ≥32 µg/ml) isolates from patient CF11 encode an in-frame R18-R19-A20 duplication in Ptr1 that affects the variable N-terminal coenzyme binding region (T-G-X_3_-G-X-G) (36). Similarly, the latter CF6 isolate has a frameshift mutation at residue 20 of Ptr1, which results in truncation (Table S3). Ptr1 is a homologue of FolM, one of two dihydrofolate reductases in bacteria (37). Loss of *folM* in a transposon-mutagenized *Escherichia coli* library has been shown to benefit bacterial growth in the presence of TMP and sulfamonomethoxine due to the upregulation of the second dihydrofolate reductase gene, *folA*, which counteracts the effect of the drugs (38). Further, mutations in the active site of FolA can confer resistance to TMP (39). Interestingly, this isolate also encodes an L30F mutation in a FolA-like dihydrofolate reductase (*BPSL2476*; Table S3). The equivalent amino acid was also mutated in laboratory-induced TMP-resistant *E. coli* populations (40) and is in close proximity to the substrate binding site (41). This isolate therefore has three putative mechanisms (i.e., altered BpeT, truncated Ptr1, and altered FolA) that may, either singly or in combination, contribute to the high TMP/SMX resistance.

In CF9, the molecular basis for TMP/SMX resistance remains elusive as there was no common mutation that could be linked to resistance in the four resistant isolates. As there are at least three subpopulations in CF9, resistance has likely occurred multiple times through multiple mechanisms. Four isolates had a deleterious, nonsynonymous SNP in *ptr1* that caused a W116R substitution (Table S4); however, only three were resistant to TMP/SMX (MIC of ≥32 µg/ml). It is thus plausible that resistance in these isolates has emerged as a consequence of two separate, and possibly synergistic, mutations. The fourth TMP/SMX-resistant isolate encoded a nonsynonymous SNP within the AmrR TetR-type regulator (*BPSL1805*), which controls the expression of the aminoglycoside and macrolide resistance pump AmrAB-OprA (42), and two nonsynonymous SNPs, causing a deleterious mutation in 5,10-methylenetetrahydrofolate reductase, encoded by *metF* (*BPSL3288*). A dysfunctional MetF may skew production toward dihydrofolate instead of tetrahydrofolate, potentially outcompeting TMP as the substrate of dihydrofolate reductase. Together with increased AmrAB-OprA efflux, dysfunctional MetF may create the high-level resistance seen in this isolate. Further functional work is ongoing in our laboratory to define the precise TMP/SMX resistance mechanisms in the CF9 isolates.

### Doxycycline resistance in patients CF9 and CF11.

In addition to TMP/SMX resistance, two midpoint isolates from CF9 developed resistance (MIC of 48 μg/ml) or decreased susceptibility (MIC of 6 μg/ml) toward doxycycline. The latter isolate of CF11 also had decreased susceptibility (MIC of 8 μg/ml) toward doxycycline. Although RND efflux systems are known to cause efflux of doxycycline to some extent (43), the specific mechanism causing the increased tolerance and resistance in these isolates is not yet known. The genome sequences generated for these isolates will be useful for helping to elucidate the molecular basis for this resistance in future studies.

### Decreased meropenem susceptibility in patients CF8, CF9, and CF11.

To date, only one report describing the development of imipenem resistance in *B. pseudomallei* (8) and three documented cases of decreased meropenem susceptibility (44) have been published. The current clinical guidelines for carbapenem resistance in *B. pseudomallei* detail breakpoint MIC values only for imipenem (≤4, 8, and ≥16 μg/ml for sensitive, intermediate, and resistant isolates, respectively) and not for meropenem (45, 46). We therefore used a MIC of ≥3 μg/ml to denote decreased meropenem susceptibility. In our study, we observed a decrease in susceptibility to meropenem in isolates from patients CF8, CF9, and CF11 (Table S5; range, 3 to 4 μg/ml). The molecular mechanisms for this decreased susceptibility are currently unknown. The clinical implications of raised meropenem MICs have not yet been fully investigated but are likely significant given that this drug is commonly used to treat the most severe and life-threatening melioidosis cases (20).

### Molecular basis for ciprofloxacin resistance in *B. pseudomallei*.

In addition to showing resistance to TMP/SMX and CAZ, the latter patient CF6 isolate was resistant to ciprofloxacin (MIC of ≥32 μg/ml; Table S3), a fluoroquinolone antibiotic commonly administered to patients with CF for treatment of exacerbations and early eradication of *P. aeruginosa* infections (47). Patient CF6 was treated with ciprofloxacin in an attempt to eradicate both *P. aeruginosa* and *B. pseudomallei* infections. Our genomic analysis revealed that a Y77S amino acid change in DNA gyrase subunit A (GyrA) is a putative mechanism for this ciprofloxacin resistance. Using stepwise fluoroquinolone selection of *B. pseudomallei* laboratory mutants, Viktorov and colleagues observed GyrA mutations T83I, G81C, and D87Y in resistant strains (48). Thus, ciprofloxacin resistance in *B. pseudomallei* is likely encoded by multiple missense *gyrA* mutations, and here we show that the Y77S mutation may also confer ciprofloxacin resistance, although functional confirmation of its role was not part of the present study.

### Mutation of secretion systems and their regulators attenuates virulence.

Many Gram-negative bacterial pathogens encode secretion systems that facilitate the injection of various virulence effector molecules directly into the host cell cytoplasm, enabling them to bypass the extracellular environment and thus host defenses. *B. pseudomallei* encodes several secretion systems, including three type III secretion systems (T3SS-1 to T3SS-3) and six type VI secretion systems (T6SS-1 to T6SS-6), of which only T3SS-3 and T6SS-1 are essential for *in vivo* virulence in mammals (49, 50). In *B. pseudomallei*, deletion or inactivation of any T3SS-3 or T6SS-1 machinery component causes virulence attenuation (49, 50).

Previous studies have shown that the T3SS-3 and T6SS-1 secretion systems and their regulators can be mutated in chronically infecting *B. pseudomallei* isolates. CF isolate Bp1651 carried two SNPs in *virA* (*BPSS1495*) that cause A475T and A606G missense mutations (8). Similarly, 37-month and 139-month chronic-carriage isolates collected from a patient infected with *B. pseudomallei* since 2000 carried a 1-bp indel in *virG*, resulting in aberrant elongation of VirG; the 139-month isolate had also shed key T3SS-3 “injectisome” genes (14). VirAG (*BPSS1494* and *BPSS1495*) is a two-component system that activates T6SS-1 (51). In keeping with these earlier findings, we observed several mutations affecting T3SS-3 and T6SS-1 loci in our CF isolates. In the latter isolate from CF6, the T3SS-3 inner membrane transport component *bsaW* (*BPSS1537*) has a D175G missense mutation, and in the latter isolate from CF1 and in both isolates from CF11, we observed an in-frame L49 duplication in VirA. Although the functional consequence of these mutations is not known, the observation that four chronically infected patients possessed mutations in *virAG* adds further weight to the hypothesis that this virulence factor control system is disadvantageous for long-term *B. pseudomallei* survival in the human host.

### Convergent evolution in fatty acid biosynthesis genes.

Phospholipids are an integral part of the lipid bilayer in Gram-negative bacteria, particularly the inner cell membrane (52), of which fatty acids are a major component. *De novo* fatty acid biosynthesis is the main determinant of the biophysical properties of the membranes since there is no alternative pathway in most bacteria (53). We identified several convergent nonsynonymous mutations affecting the FabF and FabG fatty acid elongation proteins. The latter isolate from patient CF6 possesses an N243S substitution in FabG (encoded by *BPSL2440*) and a G202S substitution in FabF (encoded by *BPSL2438*). FabF is also mutated in CF1, resulting in a G202A substitution, and FabG is mutated in CF11, leading to a T13A amino acid change. These mutated isolates have likely arisen as a response to antibiotic treatment or other environmental cues by changing the production or composition of their fatty acids, thereby altering membrane fluidity and permeability. Chronic isolates of *B. cenocepacia* have been shown to have a higher proportion of unsaturated fatty acids in their membranes compared to earlier isolates (54), and in chronic *B. multivorans* infections, several genes involved in fatty acid metabolism have been mutated (17). Although further functional studies are needed to elucidate the precise effect of these mutations in the CF-adapted *B. pseudomallei* isolates, they represent a potentially promising target for directed therapies due to their convergent nature in the *Burkholderiaceae*.

### Reductive evolution plays a role in *B. pseudomallei* adaptation in the CF lung.

Adaptation of environmental bacteria to the human host commonly results in shedding of nonessential genes from the genome (i.e., reductive evolution) or, occasionally, uptake of favorable genetic material from other microbes (55), thereby reducing metabolic expenditure and increasing the selective advantage of the bacterium (56). Gene reduction has been documented in *P. aeruginosa* strains from chronic CF lung infections (57) and in chronic-carriage *B. pseudomallei* isolates (14).

In this study, we found no evidence of DNA uptake in any of the CF pairs (data not shown). However, longitudinally collected isolates from two of the seven patients underwent reductive evolution. The latter isolate from CF8 encodes a 35-kb deletion on chromosome II encompassing *BPSS1130* to *BPSS1160* (Table S3). This deletion affected one of the two copies of the membrane-bound NarIJHG respiratory nitrate reductase system (encoded by *BPSS1156* to *BPSS1159*) and the nitrate-nitrite transporter protein NarK (*BPSS1154*). This operon is likely required for growth under hypoxic conditions rather than for anaerobic respiration since strains lacking *narG* (encoded by *BPSL2309* on chromosome I) cannot grow under anaerobic conditions, and nitrate-to-nitrite reduction is abolished during aerobic growth (58). This deletion also removed a formate-hydrogen lyase system (*BPSS1142* to *BPSS1147*), transcriptional regulators, secondary metabolism genes, additional oxidative stress response proteins, and hypothetical proteins (Table S3). Of note, all genes lost in the CF8 strain are also deleted in the equine-adapted *B. pseudomallei* clone *Burkholderia mallei*, the causative agent of glanders (59), and in a long-term chronic-carriage *B. pseudomallei* infection (14), confirming that this locus is dispensable for long-term *B. pseudomallei* persistence in the mammalian host.

Reductive evolution was also seen in CF9 isolates, with all isolates obtained after the initial strain having a 3.6-kb deletion affecting part of *BPSS1632*, which encodes a nonribosomal peptide synthetase. Additionally, the 33-month isolate had a 45.5-kb deletion encompassing part of *mutS* (as discussed earlier), and the 47-month isolate had a 5-kb deletion encompassing *BPSS2021* to *BPSS2025*. Two of these genes (*BPSS2021* and *BPSS2025*) encode sphingosine-1-phosphate (S-1-P) lyase, a putative virulence factor that irreversibly cleaves S-1-P, a bioactive metabolite in mammalian cells (60). *BPSS2021* in the 55-month isolate was also affected by a frameshift mutation, leading to loss of the original stop codon (Table S3). Loss of this enzyme in two separate isolates suggests that its inactivation may be advantageous in the CF lung environment.

### Modification of capsular and lipopolysaccharide loci assists immune evasion.

*B. pseudomallei* encodes a highly immunogenic capsular polysaccharide (CPS-I), and a type II O-antigenic polysaccharide (O-PS) moiety of lipopolysaccharide (LPS), both of which are required for serum resistance and virulence (61, 62). We have previously documented a 1-bp loss-of-function indel within the CPS-I gene, *wcbR*, which occurred prior to retrieval of the first isolate and which persisted throughout the duration of the chronic-carriage infection (14). Inactivation of CPS-I early in infection likely contributed to attenuation of virulence, immune evasion, and the unusual progression to chronic carriage in this patient. Furthermore, indels altering four LPS loci (*wbiH*, *wbiI*, *oacA*, and *BPSL1120*) were observed in isolates collected latterly from this patient, suggesting that loss of LPS is critical to immune evasion and long-term persistence (14).

On the basis of this prior work, we investigated whether CPS-I or LPS genes were mutated in the seven CF patients. Only CF11 exhibited a mutation in CPS-I, with the latter isolate encoding a 1-bp deletion in *wcbA* (*BPSL2809*). WcbA, a capsular export protein, is critical for *B. pseudomallei* virulence (62). This mutation causes a frameshift at residue A434 that truncates this protein by 70 amino acids and likely abrogates CPS-I production in this strain.

LPS mutations were more common in our data set, with the latter isolates from patients CF6, CF8, and CF11 exhibiting missense mutations at various LPS loci, none of which have been previously reported. In the latter isolate from CF6, an S81F substitution in *BPSL2681* resulted in the predicted loss of function of Wzt, a protein involved in transporting O-antigen across the inner membrane. A study on *B. mallei* by Bandara (63) performed with partially deleted *wzt* mutants showed decreased growth rates *in vitro* and increased 50% lethal doses (LD_50_) compared to the wild-type results, although no alteration in serum resistance was observed, suggesting that such mutants can evade the immune system but are still fully capable of survival and persistence. A second SNP in the CF6 isolate affected the active site of *rmlA* (*BPSL2685*), which encodes glucose-1-phosphate thymidylyltransferase, an enzyme involved in type II O-PS biosynthesis (61). Although the role of this mutation is not yet known, its location within a critical residue may abrogate, or at least impair, LPS biosynthesis in this strain. An A331T mutation (encoded by *BPSL1490*) in UDP phosphate alpha-4-amino-4-deoxy-l-arabinose (UDP-Ara4N) arabinosyl transferase, an enzyme involved in LPS metabolism (64), was observed in patient CF8. While this change was predicted to be neutral, it might still affect LPS biosynthesis and thus the composition of the outer membrane. Finally, in the latter isolate from patient CF11, an F196S substitution mutation affected *BPSL1119*, the putative LPS biosynthesis gene; this mutation was predicted to be deleterious to protein function. Functional analysis of these CPS and LPS mutants in future studies will provide invaluable insights into their role in attenuating virulence and evading immune surveillance.

### Other cell membrane components mutated in longitudinal CF isolates may play a role in immune evasion.

**FIG 3:**
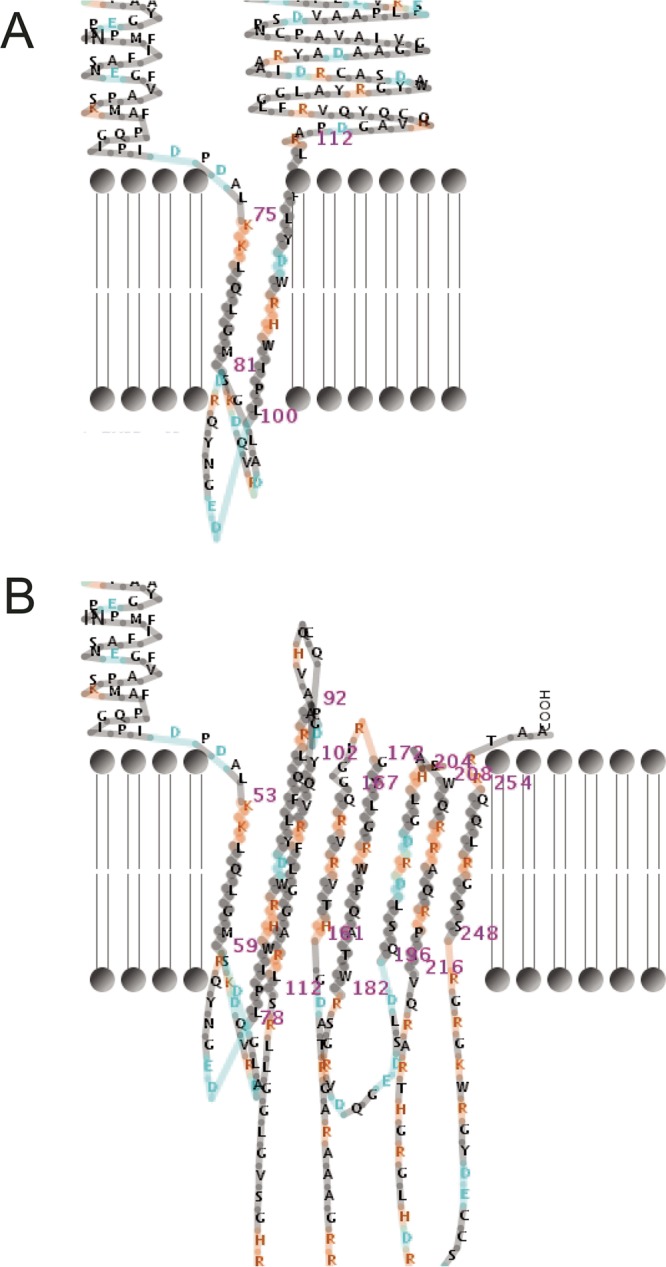
Wild-type and mutated BPSL1659. (A) Wild-type BPSL1659 conformation as seen in K96243 and MSHR5662. (B) Mutated BPSL1659 conformation in Bp1651 and MSHR5667, as modeled with PRED-TMBB posterior coding (67).

Similarly to CPS-I and LPS, OmpA is a virulence factor in *B. pseudomallei* that exhibits a high degree of immunogenicity in melioidosis patient sera (65). OmpA is an outer membrane protein that provides mechanical stability to the peptidoglycan layer (66). OmpA functions as a pore but is also involved in adhesion and immune recognition through its extracellular loops (66). The latter patient CF6 isolate has one nonsense mutation in OmpA (encoded by *BPSL2522*) that results in truncation from 224 to 217 residues. A 1-bp insertion previously described in multidrug-resistant CF isolate Bp1651 (8), which confers a frameshift at L110 in OmpA family protein BPSL1659, was also observed in the patient CF9 47-month isolate. This mutation truncates BPSL1659 by 19 residues and alters the downstream amino acid sequence, which in turn affects the protein conformation structure. Modeling showed that the wild-type BPSL1659 protein has one pair of β-sheets that span the bacterial membrane, but, following the L110 frameshift mutation, four β-sheets are predicted to span the membrane, potentially creating a more efficient transportation pore (Fig. 3) (67). This OmpA modification demonstrates yet another parallel adaptive mechanism employed by *B. pseudomallei* during chronic infection in the CF lung environment.

Peptidoglycan modifications in chronic *P. aeruginosa* CF infections have been shown to decrease innate immune activation (68). Similarly, the latter isolates from patients CF7 and CF9 encode mutations within multiple peptidoglycan synthesis genes that potentially assist in immune evasion. The latter isolate from patient CF7 has a four-residue (P42 to G45) in-frame deletion of peptidoglycan cross-linking penicillin-binding protein 1C (PBP-1c). In *B. mallei*, a four-residue in-frame deletion in PBP-1c has been suggested to play a role in immune evasion (69). In patient CF9, the 20-month and 55-month isolates have missense mutations in *murA* (encoding UDP-N-acetylglucosamine 1-carboxyvinyltransferase) and *murD* (encoding UDP-N-acetylmuramoylalanine-d-glutamate ligase), respectively. Further, the 47-month isolate has a G170D substitution in PBP3 (*BPSS1219*), a peptidoglycan-cross-linking protein. Deletion of *BPSS1219* is associated with altered morphology and increased CAZ resistance (29); however, neither phenotype was seen in this isolate. Therefore, the most probable explanation for these peptidoglycan biosynthesis alterations in the isolates from patients CF7 and CF9 is that they enhance innate immune system evasion, as has been observed in *P. aeruginosa*, rather than respond to antibiotic treatment.

### Alteration of amino acid metabolism gene pathways does not lead to auxotrophy.

Sputum generated within the CF airway provides an excellent growth medium for bacteria (70). In chronic *P. aeruginosa* infections, the amino acids in sputum serve as the major carbon source, with downregulation of anabolic pathways and upregulation of catabolic pathways leading to auxotrophy, particularly toward methionine, leucine, and arginine (70, 71). Chronic Bcc isolates also show adaptive responses in amino acid metabolism genes (17, 27, 72), and a transposon-mutagenized *B. pseudomallei* auxotroph was shown to be significantly attenuated in the murine model (73), demonstrating that such mutants can be less virulent than their wild-type counterparts.

In our previous work on *B. pseudomallei* from a chronic-carriage infection, a strain isolated 139 months after diagnosis had lost several metabolic genes encoding amino acids (14), potentially rendering this isolate auxotrophic. We also saw alterations of amino acid biosynthesis and metabolism gene pathways in isolates from patients CF6, CF9, and CF11, suggesting the potential for the evolution of amino acid auxotrophy. These mutations were predicted to affect pathways for cysteine, histidine, tryptophan, phenylalanine, tyrosine, lysine, arginine, leucine, isoleucine, and valine biosynthesis (Table S4). Auxotrophy was ruled out in the three cases on the basis of strong growth of all isolates on M63 minimal media after 5 days (results not shown). These results demonstrate the redundant nature of amino acid synthesis pathways encoded by *B. pseudomallei*.

## MATERIALS AND METHODS

Ethics approval and consent to participate in this study was obtained as previously reported (5, 7).

### Clinical history of the seven patients with CF.

The clinical history of each patient is detailed in Text S1 in the supplemental material.

10.1128/mBio.00356-17.1 TEXT S1  Clinical history of CF patients and genome assembly of MSHR0913 and MSHR5655. Download TEXT S1, DOCX file, 0.03 MB. Copyright © 2017 Viberg et al. 2017 Viberg et al. This content is distributed under the terms of the Creative Commons Attribution 4.0 International license.

### Culturing, DNA extraction, WGS, and genomic analysis.

Clinical samples were collected during normal care and cultured using standard clinical CF microbiology techniques. A single colony of each positive culture was subcultured from selective agar and identified as *B. pseudomallei* using previously outlined biochemical and molecular methods (11, 74). A single colony from each subculture was thereafter randomly chosen and frozen in 15% glycerol to create a working library. Isolates from Queensland and New Zealand CF cases were shipped to Darwin on agar slopes. Prior to DNA extraction, isolates were first grown on Ashdown’s agar (Oxoid, Thebarton, SA, Australia), with a single colony subcultured onto chocolate agar (Oxoid). High-quality DNA was extracted from purified colonies of each isolate using a previously reported method (75). Isolates were sequenced with a paired-end NextEra library using an Illumina HiSeq 2000 platform (Illumina Inc., San Diego, CA) at either Macrogen Inc. (Geumcheon-gu, Seoul, Republic of Korea) or the Australian Genome Research Facility (Parkville, Victoria, Australia). Five of the initial isolates (MSHR5651, MSHR8436, MSHR5662, MSHR8438, and MSHR8441) have been previously assembled into improved high-quality draft genomes (76), and in the current study, initial isolates MSHR0913 and MSHR5655 were assembled into high-quality draft genomes as outlined in Text S1. The seven genomes were annotated by the NCBI Prokaryotic Genome Annotation Pipeline (PGAP) (77). Annotated genomes were imported into the SnpEff v4.1 (78) database and used as a reference for comparative genomic analyses. For all isolates, SPANDx v3.1 (79) was used to identify SNPs and indels using BWA (v 0.6.2) (80) and GATK (v 3.1) (81), with larger deletions detected using BEDTools (v 2.18.2) (82). Assessment of the genomic or plasmid origin of tandemly repeated loci was performed using Bandage v 0.8.1 (31). Protein function was investigated with PROVEAN (83). BEDTools was used to generate genome-wide coverage and depth data, which were subsequently visualized using the R package ggplot2 (84).

### Phylogenetic analysis.

Core genome, biallelic SNPs identified by SPANDx were used to reconstruct a whole-genome phylogeny of 168 public *B. pseudomallei* genomes and all CF genomes described in this study. For maximum parsimony, the heuristic search function of PAUP* (v4.0b10) (85) was used and, where indicated, bootstrapped over 500 iterations. ExaML was used for inferring maximum likelihood trees (86). A combined SNP-indel maximum parsimony phylogeny (24, 25) was also constructed for patient CF9 isolates using PAUP* to gain the highest possible resolution of genotypes. Tempest v1.5 (87) was used to perform root-to-tip regression analysis of CF9 phylogenies.

### Multilocus sequence typing (MLST).

Sequence types (STs) were determined for all strains examined in this study using either conventional MLST (88) or (for sequences directly collected from assembled genomes) BIGSdb (89). All MLST profiles have been submitted to the *B. pseudomallei* MLST database (http://pubmlst.org/bpseudomallei/).

### MIC determination.

MICs were determined using Etests (bioMérieux, Baulkham Hills, NSW, Australia) following the manufacturer’s instructions. Data were assessed using Clinical and Laboratory Standards Institute M7 and M45 guidelines for determination of susceptibility or resistance in *B. pseudomallei* (45, 46). Multidrug resistance was defined as resistance to ≥2 clinically relevant antibiotic classes.

### Quantitative real-time PCR for *penA* copy number determination.

The copy number of *penA*, a β-lactamase gene, was determined using relative quantification with primers penAP167S_ForWT and penAP167S_RevWT (30). Genomic DNA was used as the template. A highly robust assay targeting the TTS1 locus (74) was used to normalize expression values.

### Determination of amino acid auxotrophy.

Isolates from patients CF6, CF9, and CF11 were grown on Mueller-Hinton agar plates (Oxoid) at 37°C for 24 h prior to plating onto M63 agar. Growth on M63 agar was assessed at days 3, 5, and 7.

### Statistical analyses.

Intragenic bias and the expected distribution of mutations were determined as described elsewhere (18). Approximately 84% of the *B. pseudomallei* genome is intragenic as determined on the basis of information available for 11 strains (90).

### Accession number(s).

All WGS data generated in this study are publicly available at the NCBI (BioProject number PRJNA272882). Table S2 in the supplemental material gives details of accession numbers for specific genome assemblies and raw DNA sequences.
