# Supplementary figures and images for: Within-Host Evolution of Burkholderia pseudomallei during Chronic Infection of Seven Australasian Cystic Fibrosis Patients
Source: mBio. 2017 Apr 11;8(2):e00356-17. doi: 10.1128/mBio.00356-17 (PMC5388805; doi:10.1128/mBio.00356-17)

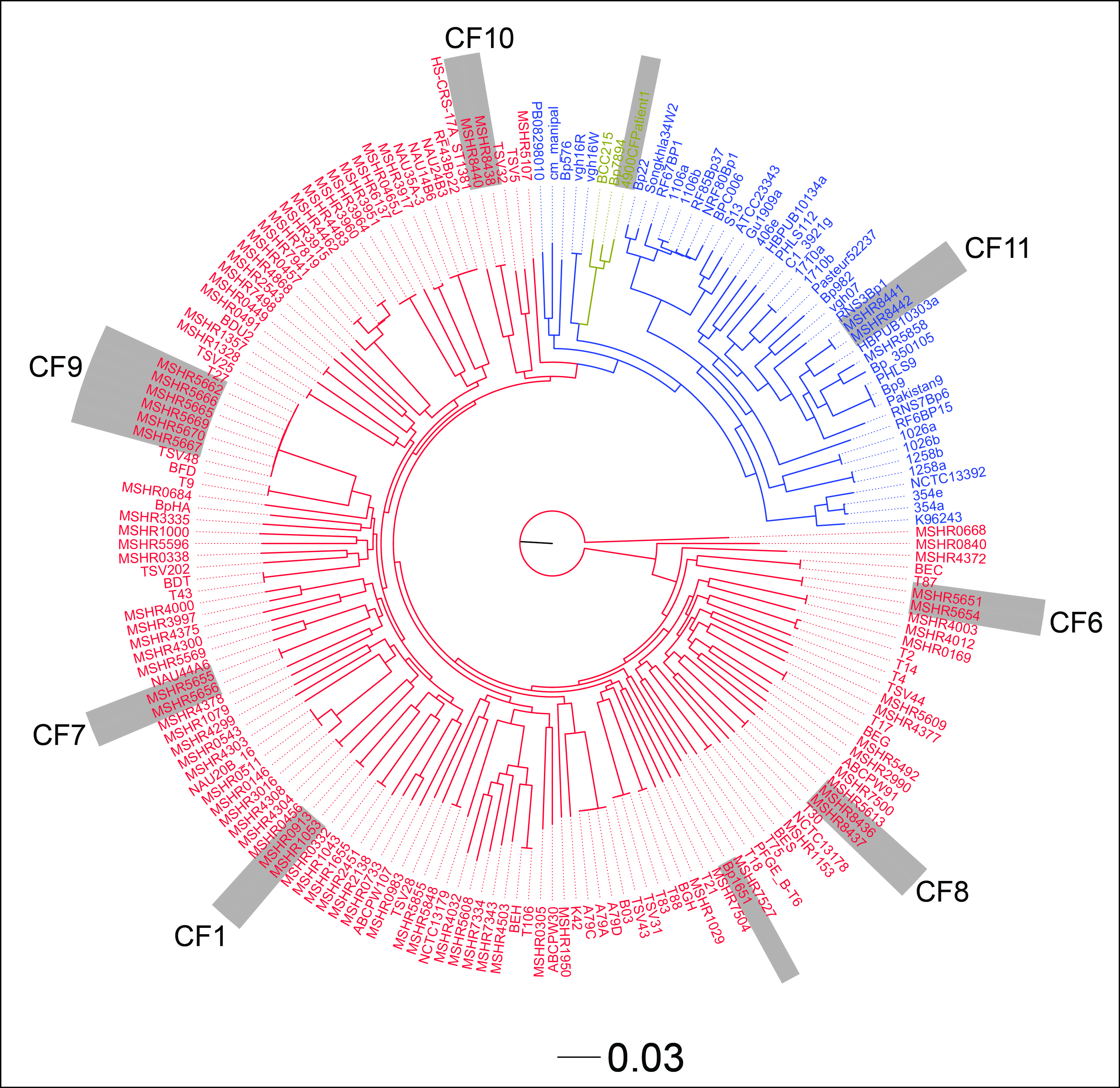

Supplement: FIG S1 [file mbo002173266sf1.jpg]

**A**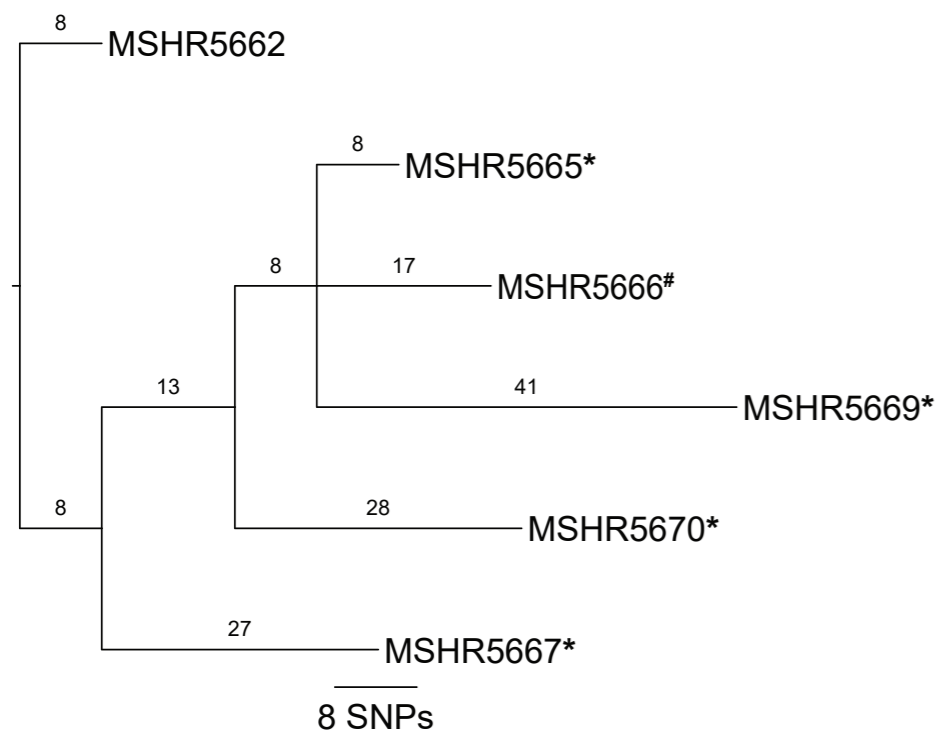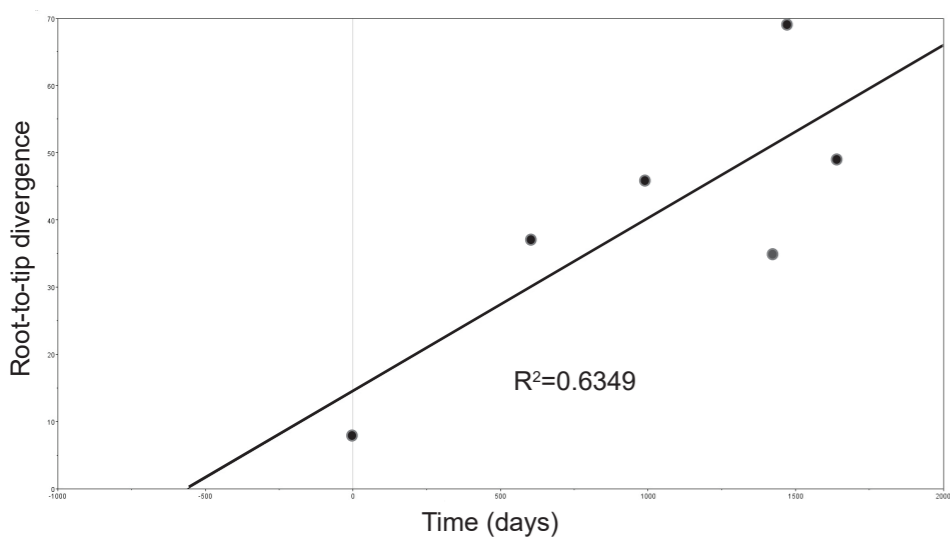**B**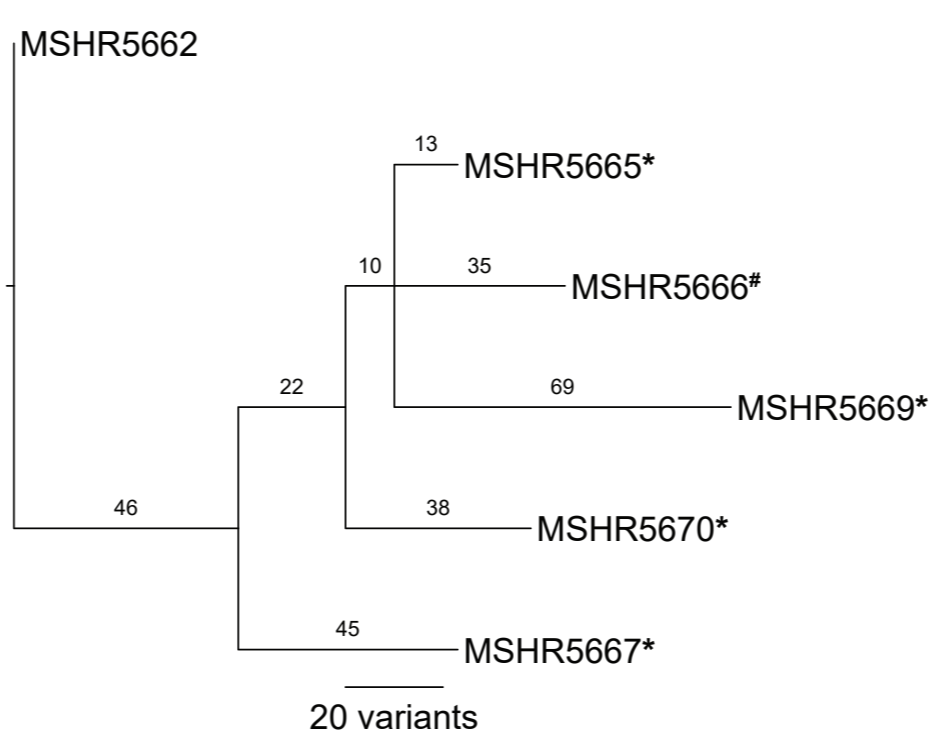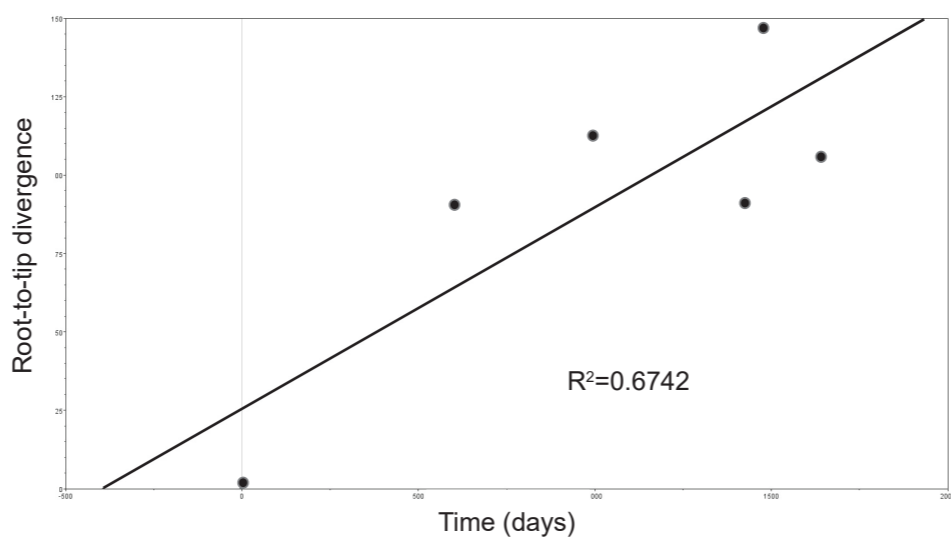**C**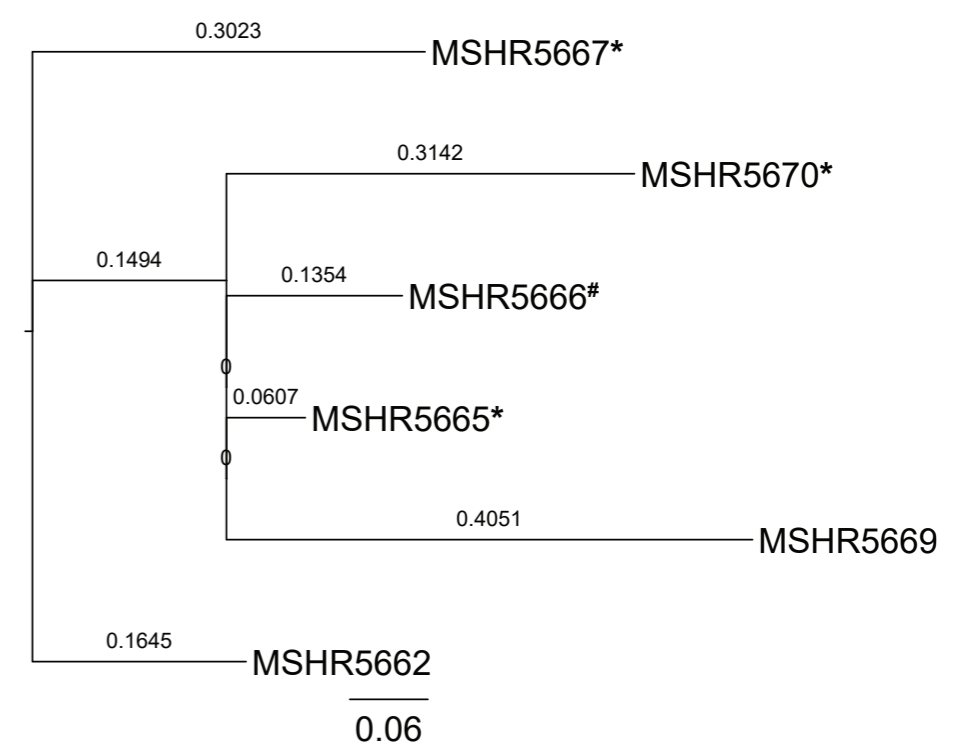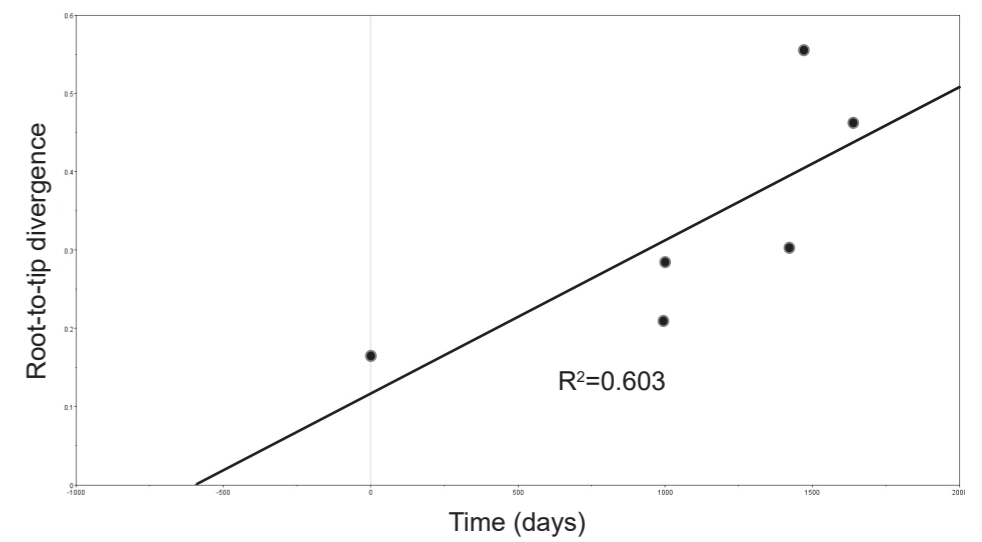

Supplement: FIG S2 [file mbo002173266sf2.pdf]
