# Supplementary material for: Within-Host Evolution of Burkholderia pseudomallei during Chronic Infection of Seven Australasian Cystic Fibrosis Patients
Source: mBio. 2017 Apr 11;8(2):e00356-17. doi: 10.1128/mBio.00356-17 (PMC5388805; doi:10.1128/mBio.00356-17)
Supplement: TEXT S1 [file mbo002173266s1.docx]

**Text S1. Clinical history of CF patients and genome assembly of MSHR0913 and MSHR5655.**

**Clinical history of the CF patients**

**Cystic fibrosis patient 1 (CF1):** CF1 was an 8.5 year-old male when *B. pseudomallei* was first detected in a sputum sample in a New Zealand clinic in 1999 (MSHR0913; Table 1) (1). CF1 had moved from Darwin, Australia, 3.5 years prior. Given that *B. pseudomallei* has never been retrieved from the environment in New Zealand, CF1 was likely infected whilst residing in Darwin. The patient remained clinically stable for six months before experiencing an acute deterioration in pulmonary function for which he was treated with ceftazidime and tobramycin. CF1 responded well to treatment but relapsed when therapy was discontinued. A further 17 weeks of ceftazidime and meropenem did not clear *B. pseudomallei* from the sputum and the patient remained unwell. He was desensitised to trimethoprim/sulfamethoxazole (TMP/SMX) due to previous *in vitro* sensitivity to this antibiotic, and was then treated with oral TMP/SMX. Despite further treatments directed toward *B. pseudomallei*, eradication was not successful. A further isolate, MSHR1053, was collected 13 months after MSHR0913*.* Co-pathogens in CF1’s lungs included *P. aeruginosa* and *Aspergillus fumigatus* (1, 2). The patient passed away in 2004 at 13 years of age.

**Cystic fibrosis patient 6 (CF6):** CF6 was a 21 year-old male when *B. pseudomallei* (MSHR5651) was first detected in his sputum in 2000, with the last isolate, MSHR5654, obtained 27 months after diagnosis. Treatment during this time included ceftazidime and meropenem administration for 56 days. The patient deteriorated and was temporarily stabilised with high-dose TMP/SMX before deteriorating again. Improvement was seen when treated with amoxicillin-clavulanate (AMC), although attempts to eradicate *B. pseudomallei* using tetracycline and ciprofloxacin, individually or in combination with TMP/SMX, were not successful. Co-pathogens in the lungs were *P. aeruginosa, S. aureus* and Bcc species (3). The patient experienced a rapid decline and died shortly after MSHR5654 was isolated.

**Cystic fibrosis patient 7 (CF7):** CF7 was a 38 year-old male when *B. pseudomallei* (MSHR5655) was first detected in sputum in 2007, with the most recently available isolate, MSHR5656, obtained 117 days after diagnosis. CF7 had an ongoing *B. pseudomallei* infection until late 2012, during which time he has experienced a significant decline in lung function and was subsequently listed for lung transplantation. Between six and twelve months after MSHR5655 was isolated, eradication was attempted with ceftazidime and TMP/SMX for 14 days, followed by TMP/SMX for 84 days, but was not successful initially although appeared to spontaneously clear the organism. Co-pathogens in this patient’s lungs include *P. aeruginosa*, *S. aureus* and *Aspergillus terreus* (2)**.** The two *B. pseudomallei* isolates available for this study were collected prior to eradication being attempted. In September 2016 he received a lung transplant, and to date, two bronchoscopy samples have failed to yield *B. pseudomallei*, nor was it identified in the explanted lung samples.

**Cystic fibrosis patient 8 (CF8):** CF8 was a 14 year-old male when *B. pseudomallei* (MSHR8436) was first detected in his sputum in 2001, with the most recent isolate available for this study, MSHR8437, obtained 46 months after diagnosis. Similar to CF7, this patient has had an ongoing infection for the past 15 years with a progressive decline in lung health. CF8 also has chronic infections with *P. aeruginosa* and *S. aureus* in his lungs (2, 3). There have not been any attempts to eradicate *B. pseudomallei* and the patient has declined intravenous antibiotics for addressing pulmonary exacerbations.

**Cystic fibrosis patient 9 (CF9**): CF9 was a 21 year-old male when *B. pseudomallei* was first isolated from his sputum in 1987, and was thought to have been infected in northern Queensland, Australia. The first isolate obtained for this study, MSHR5662, was collected in 2000. He has had several courses of intravenous ceftazidime and/or meropenem with oral TMP/SMX to alleviate pulmonary exacerbations. In between exacerbations, he has been maintained on TMP/SMX or tetracycline. In 2004, CF9 had a bilateral lung transplant. Despite the transplant, CF9 continues to harbour *B. pseudomallei* and *P. aeruginosa* (2, 3), and has normal and stable lung function as of July 2016. Six sequential isolates were obtained from CF9 for this study (Table 1), including two isolates collected prior to the lung transplant (MSHRs 5665 and 5666), and two isolates collected two days (MSHR5667) and two months (MSHR5669) post-transplantation (i.e. 47 and 49 months post-MSHR5662). The most recent isolate, MSHR5670, was collected 55 months after the study’s first isolate, or 7 months post-transplantation.

**Cystic fibrosis patient 10 (CF10**): CF10 was a 25 year-old female when *B. pseudomallei* (MSHR8438) was first identified in sputum in 2002. The last isolate in this study was obtained 10 months after diagnosis. During this time, treatment included three weeks of intravenous ceftazidime, meropenem and tobramycin to manage a pulmonary exacerbation with consideration of co-pathogens. The patient had a chronic infection lasting two years but had since cleared their *B. pseudomallei* infection. Co-infecting pathogens included *P. aeruginosa, S. aureus* and *B.* *cenocepacia* (3). The patient died from respiratory failure secondary to severe lung disease in 2011.

**Cystic fibrosis patient 11 (CF11**): CF11 was a 10 year-old female when *B. pseudomallei* was first detected in her sputum in a New Zealand clinic in 2007; however, the first isolate available for this study (when reviewed clinically in Brisbane, Australia), MSHR8441, was not obtained until 2010. Prior to *B. pseudomallei* detection, she had travelled to Southeast Asia where she is suspected to have contracted the infection. The last isolate, MSHR8442, was collected 14 months after diagnosis. Eradication was attempted approximately two months after diagnosis with intravenous ceftazidime, tobramycin and AMC for 14 days. The infection did not clear and was followed with a second round of intravenous treatment of meropenem, ceftazidime and AMC for 84 days, followed by long-term oral treatment with TMP/SMX and doxycycline. Both isolates in this study were obtained after the eradication attempts. Co-pathogens of CF11’s lungs were *P. aeruginosa* and *S. aureus* (2). The patient had an accelerated decline and passed away at 16 years of age, six years after first detection of *B. pseudomallei*.

**Genome assembly of MSHR0913 and MSHR5655**

The initial isolates MSHR0913 and MSHR5655 were assembled into draft genomes using the MGAP v1.0 (<https://github.com/dsarov/MGAP---Microbial-Genome-Assembler-Pipeline>). Contigs were re-ordered relative to the closed Australian *B. pseudomallei* genome, MSHR1153 (4), using Mauve v2.4.0 (5). Contig joins were manually checked for synteny using BLAST and joined if appropriate. Assembled genomes were further corrected for false-positive single-nucleotide polymorphisms and insertions/deletions by mapping the Illumina reads to the reference using default settings in SPANDx v3.1 (6).

1. **Holland DJ, Wesley A, Drinkovic D, Currie BJ.** 2002. Cystic fibrosis and *Burkholderia pseudomallei* infection: an emerging problem? Clin Infect Dis **35**:e138-e140.

2. **Geake JB, Reid DW, Currie BJ, Bell SC, Melioid CFI.** 2015. An international, multicentre evaluation and description of *Burkholderia pseudomallei* infection in cystic fibrosis. BMC Pulm Med **15**:116.

3. **O’Carroll M, Kidd T, Coulter C, Smith H, Rose B, Harbour C, Bell S.** 2003. *Burkholderia pseudomallei*: another emerging pathogen in cystic fibrosis. Thorax **58**:1087-1091.

4. **Johnson SL, Baker AL, Chain PS, Currie BJ, Daligault HE, Davenport KW, Davis CB, Inglis TJ, Kaestli M, Koren S, Mayo M, Merritt AJ, Price EP, Sarovich DS, Warner J, Rosovitz MJ.** 2015. Whole-genome sequences of 80 environmental and clinical isolates of *Burkholderia pseudomallei*. Genome Announc **3**.

5. **Rissman AI, Mau B, Biehl BS, Darling AE, Glasner JD, Perna NT.** 2009. Reordering contigs of draft genomes using the Mauve aligner. Bioinformatics **25**:2071-3.

6. **Sarovich DS, Price EP.** 2014. SPANDx: a genomics pipeline for comparative analysis of large haploid whole genome re-sequencing datasets. BMC Res Notes **7**:618.
